# Molecular Characteristics and Biochemical Functions of VpPR10s from *Vitis pseudoreticulata* Associated with Biotic and Abiotic Stresses

**DOI:** 10.3390/ijms151019162

**Published:** 2014-10-22

**Authors:** Lan Wang, Jinyu Wei, Ying Zou, Keyao Xu, Yuejin Wang, Lu Cui, Yan Xu

**Affiliations:** 1 State Key Laboratory of Crop Stress Biology in Arid Areas, Northwest A&F University, Yangling 712100, Shaanxi, China; E-Mails: wanglan0427@163.com (L.W.); irene0908@126.com (J.W.); zouyingqnh@126.com (Y.Z.); xukeyao728@sina.com (K.X.); wangyj@nwsuaf.edu.cn (Y.W.); 2 College of Horticulture, Northwest A&F University, Yangling 712100, Shaanxi, China; 3 Key Laboratory of Horticultural Plant Biology and Germplasm Innovation in Northwest China, Ministry of Agriculture, Yangling 712100, Shaanxi, China; 4 College of Food Science Engineering, Northwest A&F University, Yangling 712100, Shaanxi, China

**Keywords:** *Vitis pseudoreticulata*, pathogenesis-related protein 10, nuclease activity, anti-fungal activity, abiotic stresses

## Abstract

Grapes are one of the world’s oldest and most important fruit crops. They are of high economic value in many countries, but the susceptibility of the dominant winegrape species *Vitis vinifera* to fungal disease is a significant problem. The Chinese wild grape species are a rich source of disease-resistance genes and these can be used to discover how disease resistance in *V. vinifera* grapevines might be enhanced. Pathogenesis-related (PR) 10 proteins are involved in the disease-response. Here, we use the genomic DNA of the Chinese wild species *Vitis pseudoreticulata* accession “Baihe-35-1” as the template to design specific primers based on *VvPR10s* sequences. We used overlap extension PCR to obtain the sequences: *VpPR10.4*, *VpPR10.6*, *VpPR10.7* and *VpPR10.9*. The coding sequences of the *VpPR10s* were then cloned into the pGEX-4T-1 vector. The purified proteins VpPR10.4, VpPR10.6, VpPR10.7 and VpPR10.9 were used to analyse nuclease activity. Meanwhile, functional analysis of VpPR10s under different biotic and abiotic stresses was carried out to further clarify the disease-resistance mechanisms of the Chinese wild grapevine *VpPR10* genes. The analysis of protein structure indicates that VpPR10.4 and VpPR10.7 had the P-loop domain and the Bet v 1 motif, which are a consistent feature of plant PR10. However, there was no P-loop domain or Bet v 1 motif in VpPR10.9 and we could not find the Bet v 1 motif in VpPR10.6. The results of the nuclease activity assay and of the functional analyses of VpPR10s under different biotic and abiotic stresses also confirm that VpPR10.4 and VpPR10.7 proteins have marked RNase, DNase, anti-fungal activities and respond to abiotic stresses. The VpPR10.6 and VpPR10.9 proteins do not have these activities and functions.

## 1. Introduction

Grapes have been cultivated for a very long time and, today, are one of the world’s more important fruit crops. However, most cultivars of the most commonly cultivated species, the European grapevine *Vitis vinifera*, are highly susceptible to the disease powdery mildew caused by the fungus *Erysiphe necator* [1,2,3]. Powdery mildew causes substantial reductions in harvest yield and also in the quality of the resulting wine. The prevention and cure of the fungal diseases of grapevine are still managed largely through agrichemicals [4]. The use of many of these is not sustainable in the long term with toxic build up of heavy metals (principally copper) in the soil. Nor are their residues desirable in the processed product. Therefore, for many years one of the key goals of grapevine breeders has been to develop *V. vinifera* cultivars having enhanced disease resistance through hybridization with other, more disease-resistant species and by genetic modification [5].

The Chinese flora contains a rich germplasm resource with a number of wild grapevine species, many of which carry genes conferring strong disease resistance. For example, the Chinese wild grapevine *Vitis pseudoreticulata* accession “Baihe-35-1” has relatively highly resistance to a number of fungi, and especially to *E. necator* [6,7]. These wild grapevine germplasm resources offer the opportunity to mine novel disease-resistance genes and so accelerate the genetic improvement of our existing *V. vinifera* germplasm resources.

Plant pathogenesis-related (PR) proteins were first discovered in tobacco leaves infected by tobacco mosaic virus (TMV) [8]. PR proteins are usually induced by pathogen infection or by pathological conditions, but they do not accumulate in healthy plants. Hence, they play various roles in improving the defensive responses of plants to pathogen attack [9].

PR proteins are usually encoded by multiple genes and have been grouped into 17 families, based on the similarity of their amino acid sequences, structures, serological relationships and biological activities [8,9,10]. Most are extracellular proteins but some are localised intracellularly in the vacuole. In contrast, the PR10 proteins are the only ones residing in the cytoplasm. PR10 proteins were first discovered in cultured parsley cells after treatment with an elicitor [11]. Generally, PR10 proteins are slightly acidic, lack a signal peptide and possess an antiprotease character. The open reading frame (ORF) of most PR10 genes is between 456 and 489 bp long, encoding 151–162 amino acids, with a molecular mass of 16–19 kDa [12]. To date, members of the PR10 family have been reported in many higher plant species of both monocots [13,14] and dicots [12,15,16,17,18]. During growth [19,20], *PR10* genes are expressed in many different tissues and organs, such as in pollen grains [15,21], flowers [15,22,23,24,25], fruits [26,27], seeds [25,28], and in the vegetative organs, roots [29,30,31,32], stems [25,33] and leaves [33,34].

PR10 proteins exist widely in higher plants. Most PR10 proteins contain a highly conservative P-loop domain, which functions as a nucleotide binding site and can activate ribonuclease activity in some PR10 proteins [35]. Mutations in PR10 conservative amino acid sites have been found in cotton, sweet potato and peanut. These mutations can result in loss of ribonuclease activity in the PR10 proteins [28,36,37]. These results suggest that conservative amino acid residues of the P-loop domain play an important role in determining the ribonuclease activity of the PR10 protein. Most PR10 proteins also contain the Bet v 1 motif [38]. It is reported that the main birch pollen allergen (Bet v 1) has RNase activity [39]. Chadha and Das (2006) isolated a *PR10* gene, encoding PR10 protein containing a P-loop domain and Bet v 1 motif from the peanut cDNA library. They also showed a relationship between the antifungal activity of PR10 protein and its RNase activity *in vitro* [37]. Moreover, it has been shown that white lupin LaPR10 [40], cotton GaPR10 [36] and chili PR10 proteins [41,42] all have ribonuclease activity and antifungal activity. Besides these plants, PR10 proteins in rice [43] and pea [44] have also been confirmed as having nuclease activity *in vitro*. The VpPR10 proteins in the Chinese wild *V. pseudoreticulata* have been show not only to have RNase and antifungal activity but also to have DNase activity [45].

PR10 proteins can have a direct affect on a pathogen by nuclease activity or through programmed cell death in the infection site. The hypersensitive response (HR) is a process through which plant cells can respond to outside biological stresses. It is characterised by the rapid death of cells in the region immediately surrounding the infection, to prevent the spread of the pathogen. It has been confirmed that the *PBZ1* gene in rice, a member of the PR10 family, is related to programmed cell death [43]. After the pathogen has been induced, programmed cell death occurs as an allergic response, and the accumulation of PBZ1 protein can also be detected. It has been clearly shown that PR10 protein can cause hypersensitive responses in plant cells, thereby resulting in programmed cell death [43,46].

The expression of PR10 in plant cells can be induced by a number of biotic and abiotic stresses. The rice *JIOsPR10* genes are highly expressed under the induction of H_2_O_2_, salicylic acid (SA) or jasmonic acid (JA) [47]. The rice *RSOsPR10* genes are induced by JA, NaCl and drought but do not respond to abscisic acid (ABA), SA and low temperature [48]. The expressions of maize PR10.1 and PR10.2 proteins can also be induced by biological stresses, such as bacterial infection. Abiotic stresses, such as H_2_O_2_, mechanical damage and freezing, can induce their expressions simultaneously [49]. RzPR10 proteins in the Tunisian grape cultivar “Razegui” are induced by salt stress and may be associated with resistance to salt stress [50].

Here, we investigate the functions of the genes *VpPR10.4*, *VpPR10.6*, *VpPR10.7* and *VpPR10.9* cloned from Chinese wild *V. pseudoreticulata* accession “Baihe-35-1”. We carried out DNase and RNase activities assays, an *in vitro* anti-fungal activity assay and functional analyses under different abiotic stresses to elucidate the disease-resistant mechanism of these *VpPR10* genes and to provide a theoretical basis for other related research.

## 2. Results

### 2.1. Sequence Alignment of VpPR10s

It was found that the family resemblance of *VpPR10s* ranged from 59.0%–79.3% after nucleotide sequence analysis. The highest similarity was 79.3% between *VpPR10.4* and *VpPR10.6*, while the lowest was 59.0% between *VpPR10.4* and *VpPR10.9*. *VpPR10.4*, *VpPR10.6*, *VpPR10.7* and *VpPR10.9* contained inserts of complete ORF of 480, 450, 477 and 486 bp, which encode peptides of 159, 149, 158 and 161 amino acid residues, having 99.4% similarity (with *VvPR10.4*), 97.6% (with *VvPR10.6*), 99% (with *VvPR10.7*) and 98.8% (with *VvPR10.9*), molecular masses of 17.34, 16.90, 17.45 and 18.41 kDa and isoelectric points of 4.99, 5.99, 4.98 and 5.45, respectively.

Most PR10 proteins contain two conservative structure domains—A P-loop domain (AA 47–55, GXGGXGXXK) and a Bet v 1 motif (AA89-121, G-[DG]-[VA]-L-x(4)-E-[SY]-[IL]-[CSATV]-[HY]-[ED]-x-[KST]-x-[VE]-x(3)-[GNDS]-G(2)-[CS]-x(2)-K-x(2)-[SK]-X-Y) [38]. The P-loop domain has a nucleotide binding site, and phosphorylation of this area suggests it may be associated with nuclease activity in some PR10 proteins [14,51].

**Figure 1:**
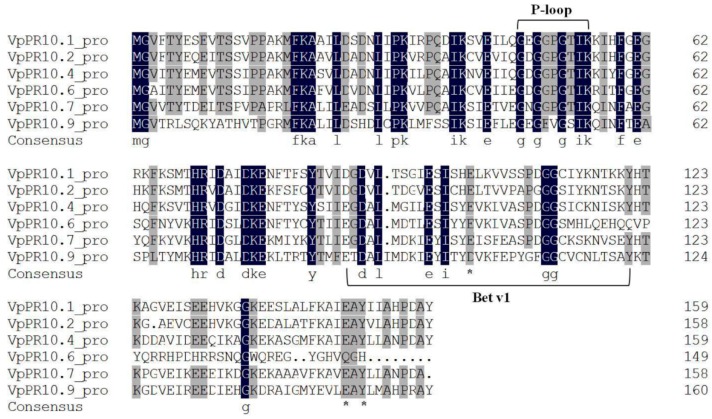
Deduced sequence alignment of PR10 proteins. Black and gray shading indicate amino acids which are identical or similar, respectively. Dashes indicate gaps introduced for optimal alignment. The P-loop and Bet v 1 are framed. The Bet v 1 motif was determined as:AA89-121,G-[DG]-[VA]-L-x(4)-E-[SY]-[IL]-[CSATV]-[HY]-[ED]-x-[KST]-x-[VE]-x(3)-[GNDS]-G(2)-[CS]-x(2)-K-x(2)-[SK]-X-Y and the P-loop domain as: AA 47-55, G-X-G (2)-X-G-X(2)-K. (*) marks the amino-acids implicated in possible ribonuclease activity (E102, E149 and Y151).

A protein motif analysis was carried out among the amino acid sequences coded by the four *VpPR10* genes. Figure 1 shows that, except for VpPR10.9, the amino acids 47–55 of the VpPR10s corresponded to the P-loop conservative structure domain. In the analysis of protein structure, it is predicted that amino acids 89–121 of VpPR10.4 and VpPR10.7 correspond to the Bet v 1 motif. However, this Bet v 1 motif was not found in the amino acid sequences of VpPR10.6 and VpPR10.9. Amino acid sequences of VpPR10.4 and VpPR10.7 contained characteristic amino acid sites of nuclease activity, E102, E149 and Y151. Nevertheless, E102 was replaced by D102 in the amino acid sequence of VpPR10.9, while E149 and Y151 were mutated to Q149 and H151 in VpPR10.6.

### 2.2. Phylogenetic Analyses of VpPR10s

**Figure 2:**
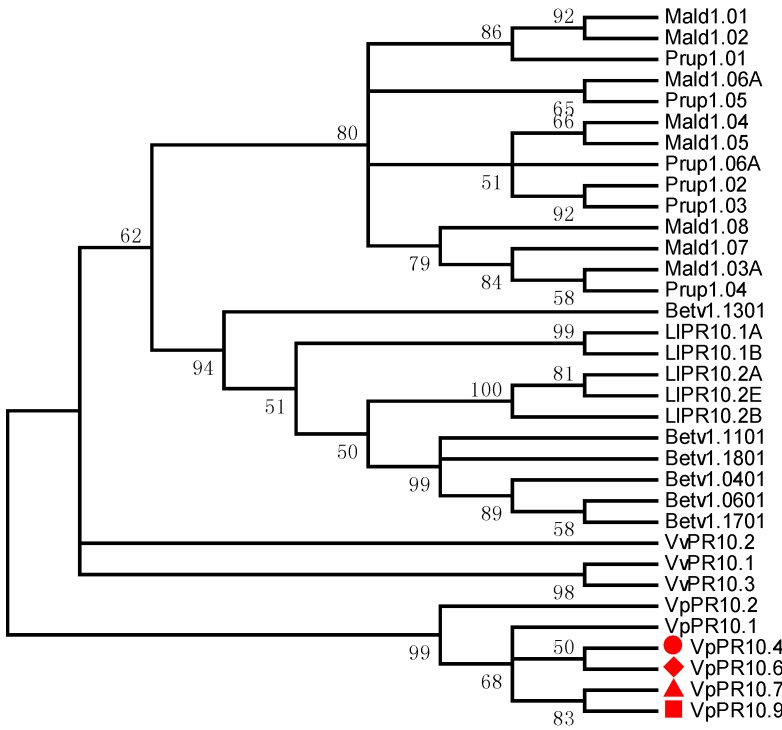
Phylogenetic relationships of the deduced amino acids in VpPR10s, and other reported PR10 proteins. GenBank accession numbers are: *Betula pendula*: Betv1.0401 (CAA54482), Betv1.0601 (CAA54484), Betv1.1101 (CAA54694), Betv1.1301 (CAA54696), Betv1.1701 (CAA96539) and Betv1.1801 (CAA96540); *Lupinus luteus*: LlPR10.1A (CAA56298), LlPR10.1B (CAA56299), LlPR10.2A (AAF77633), LlPR10.B (AAF77634) andLlPR10.2E (AAP37978); *Malus domestica*: Mald1.01 (AAX18288), Mald1.02 (AAX18291),Mald1.03A (AAX18313), Mald1.04 (AAX18294), Mald1.05 (AAX18296), Mald1.06A (AAX18299), Mald1.07 (AAX18307) and Mald1.08 (AAX18310); *Prunus persica*: Prup1.01 (ACE80940), Prup1.02 (ACE80942), Prup1.03 (ACE80944), Prup1.04 (ACE80946), Prup1.05 (ACE80948) and Prup1.06A (ACE80952); *Vitis vinifera*: VvPR10.1 (AJ291705), VvPR10.2 ( AJ291704) and VvPR10.3 (EU379313).

To further our understanding of the evolutionary location and genetic relationships of VpPR10s among the PR10 protein family and adjacent families, MEGA4.0 software was used to research the homology and phylogenetic relationships among VpPR10.4, VpPR10.6, VpPR10.7 and VpPR10.9, and other reported PR10 proteins. The clustering results of the evolutionary tree confirmed that VpPR10.4 and VpPR10.6 were close relatives and there was close similarity between VpPR10.7 and VpPR10.9 (Figure 2).

### 2.3. Purification of Recombinant Proteins

**Figure 3:**
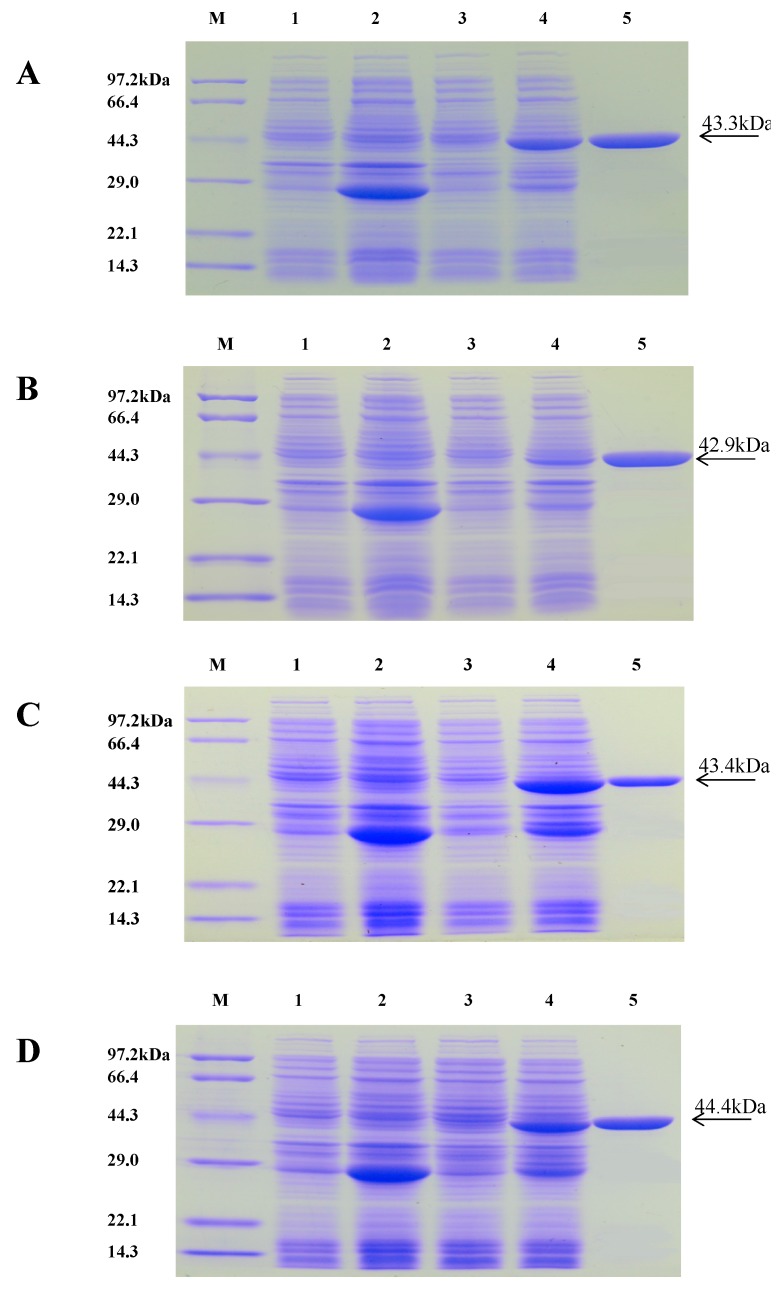
Purification of VpPR10 recombinant proteins. Lane **M**: Protein marker; Lane **1**: The total protein of null vector pGEX-4T-1 without induction (37 °C, 4 h); Lane **2**: The total protein of pGEX-4T-1 with induction (37 °C, 4 h, 0.1 mM IPTG); Lane **3**: The total protein of pGEX-VpPR10s without induction (37 °C, 4 h); Lane **4**: The total protein of pGEX-VpPR10s with induction (37 °C, 4 h, 0.1 mM IPTG); and Lane **5**: VpPR10 recombinant proteins purified with GST resin. (**A**–**D**) represent bacteria containing the recombinantexpression vector pGEX-VpPR10.4, pGEX-VpPR10.6, pGEX-VpPR10.7 and pGEX-VpPR10.9, respectively.

The recombinant VpPR10s proteins were purified with GST resin and detected with 12% concentrated gel SDS-PAGE. Single bands of purified target proteins VpPR10.4, VpPR10.6, VpPR10.7 and VpPR10.9 were observed in Lane 5, with molecular masses of 43.3, 42.9, 43.4 and 44.4 kDa, respectively (Figure 3A–D).

### 2.4. DNase and RNase Activities of VpPR10s

**Figure 4:**
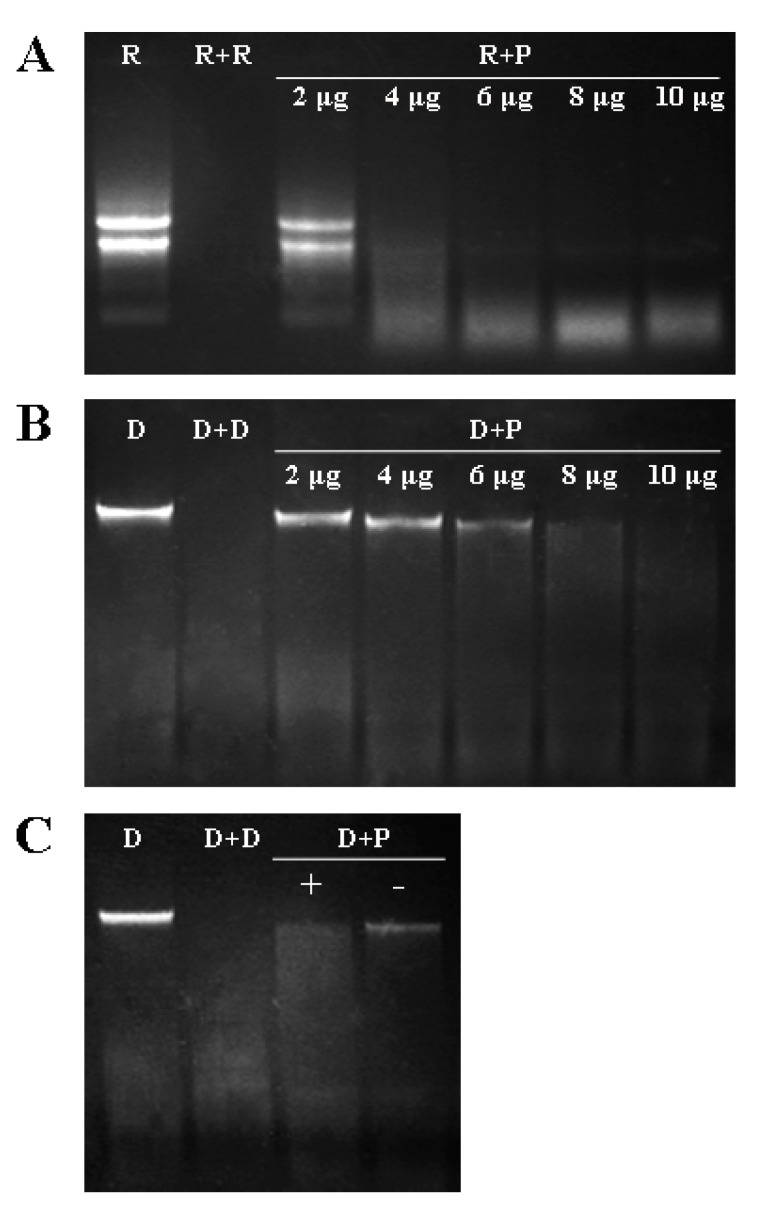
Effects of protein concentration and Mg^2+^ on nuclease activity of VpPR10.7 recombinant protein. (**A**) Effect of protein concentration on RNase activity of VpPR10.7. Lane **R**: RNA + reduced glutathione buffer (negative control); Lane **R** + **R**: RNA + RNase (positive control); Lane **R** + **P**: RNA + VpPR10.7 protein; 2, 4, 6, 8 and 10 μg represent different protein concentrations; (**B**) Effect of protein concentration on DNase activity of VpPR10.7. Lane **D**: DNA + reduced glutathione buffer (negative control); Lane **D** + **D**: DNA + DNase (positive control); Lane **D** + **P**: DNA + VpPR10.7 protein; and (**C**) Effect of Mg^2+^ on DNase activity. “+”: with 2.5 mM MgCl_2_; “–”: without 2.5 mM MgCl_2_.

In this study, the effects of protein concentration and Mg^2+^ on nuclease activity of VpPR10.7 recombinant protein were carried out with the incubation of 0, 2, 4, 6, 8 and 10 μg VpPR10.7 mixed with 100 ng·μL^−1^ “Baihe-35-1” leaf total RNA or genomic DNA for 30 min at 37 °C. The nuclease experiments showed that the degradation of “Baihe-35-1” RNA or gDNA incubated with 4–8 μg target protein was significantly observed, and 4 μg recombinant protein could thoroughly degrade total RNA in the reaction system with 30 min incubation (Figure 4A). Meanwhile, 8 μg VpPR10.7 recombinant protein was enough to degrade total “Baihe-35-1” gDNA for 30 min incubation (Figure 4B), and the 2.5 mM Mg^2+^ in the reaction mixtures could enhance the DNase activity of VpPR10.7. Based on the nuclease experimental results, 8 μg purified recombinant proteins were used to perform subsequent RNase and DNase activities assays.

**Figure 5:**
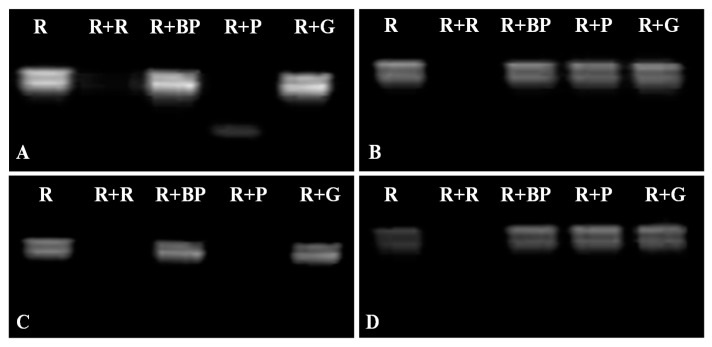
RNase activities of VpPR10 proteins. (**A**–**D**) represent the recombinant proteins pGEX-VpPR10.4, pGEX-VpPR10.6, pGEX-VpPR10.7 and pGEX-VpPR10.9, respectively. Lane **R**: RNA + reduced glutathione buffer (negative control); Lane **R** + **R**: RNA + RNase (positive control); Lane **R** + **BP**: RNA + boiled-VpPR10 protein (negative control); Lane **R** + **P**: RNA + VpPR10 protein; Lane **R** + **G**: RNA + GST-Tag protein (negative control).

The RNase activity analysis of VpPR10s proteins was carried out in accordance with the method of Yan *et al.* [52]. The degradation of total RNA by VpPR10s proteins was used to further investigate the RNase activities of VpPR10s. The degradation of RNA did not appear in the negative control using reduced glutathione buffer (lane R) and four boiled VpPR10 proteins (lane R + BP). The total RNA when treated with VpPR10.4 and VpPR10.7 proteins (Figure 5 lane R + P in A,C) showed obvious degradation while VpPR10.6 and VpPR10.9 proteins (Figure 5 lane R + P in B,D) did not show degradation.

**Figure 6:**
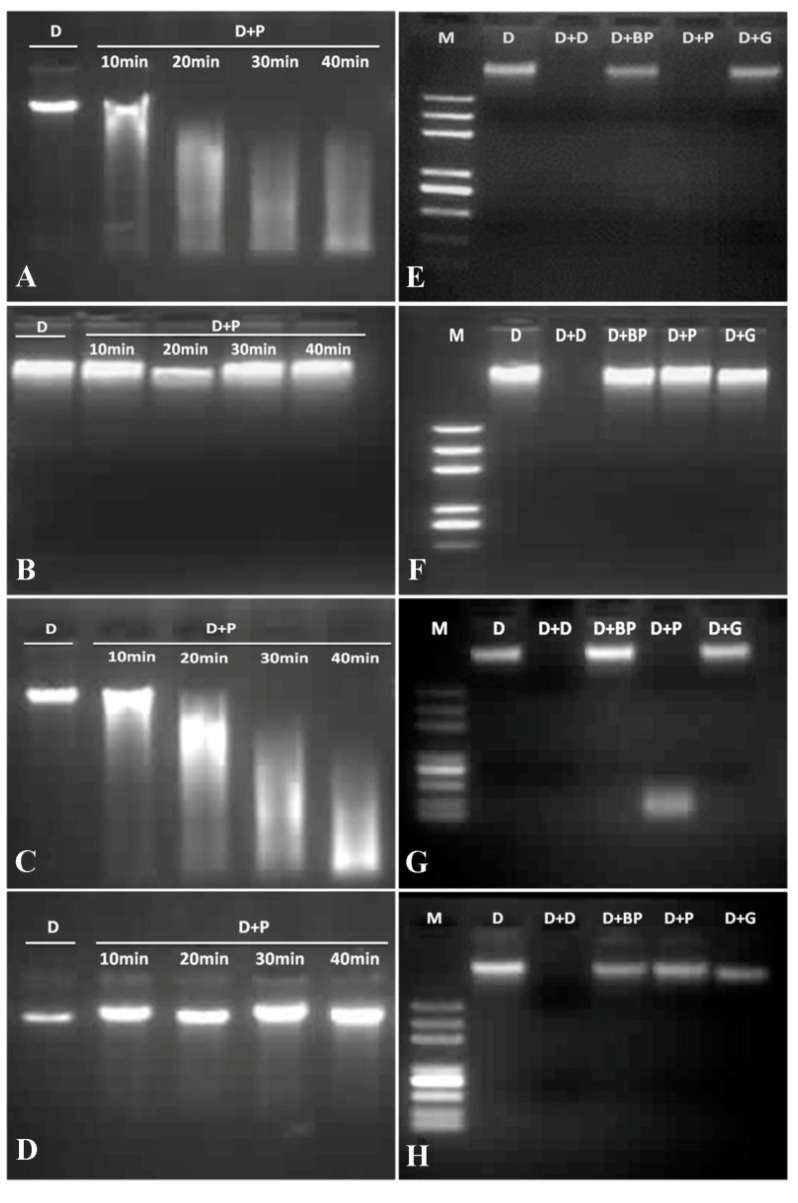
DNase activities of VpPR10 proteins. (**A**–**D**) represent the recombinant proteins pGEX-VpPR10.4, pGEX-VpPR10.6, pGEX-VpPR10.7 and pGEX-VpPR10.9, respectively.Lane **M**: DNA marker; Lane **D**: DNA + reduced glutathione buffer (negative control); Lane **D** + **P**: DNA + VpPR10 protein, 10, 20, 30 and 40 min represent different reaction times; (**E**–**H**) represent the recombinant proteins pGEX-VpPR10.4, pGEX-VpPR10.6, pGEX-VpPR10.7 and pGEX-VpPR10.9, respectively. Each reaction included 8 μg of recombinant protein. Final concentrations of gDNA and MgCl_2_ were 100 ng μL^−1^ and 2.5 mM, respectively. The mixture was thoroughly incorporated and held at 37 °C in a water bath for 30 min. Lane **D**: DNA + reduced glutathione buffer (negative control); Lane **D** + **D**: DNA + DNase (positive control); Lane **D** + **BP**: DNA + boiled-VpPR10 protein (negative control); Lane **D** + **P**: DNA + VpPR10 protein; Lane **D** + **G**: DNA + GST-protein (negative control).

The DNase activity analysis of VpPR10s proteins was carried out according to the method of Guevara-Morato *et al.* [53]. As shown in Figure 6, the various VpPR10s proteins had different levels of DNase activities. The genomic DNA did not appear to degrade with reduced glutathione buffer as the negative control. When treated with VpPR10.4 and VpPR10.7 recombinant proteins (Figure 6 lane D + P in E,G), the genomic DNA showed obvious degradation, while with VpPR10.6 and VpPR10.9 (Figure 6 lane D + P in F,H) there was almost no degradation. This shows that the degree of gDNA degradation changes with reaction time. The gDNA degraded more after 40 min when treated with VpPR10.4 and VpPR10.7 (Figure 6A,C) but did not degrade with VpPR10.6 and VpPR10.9 (Figure 6B,D).

### 2.5. In Vitro Anti-Fungal Activities of Recombinant VpPR10s Proteins

**Figure 7:**
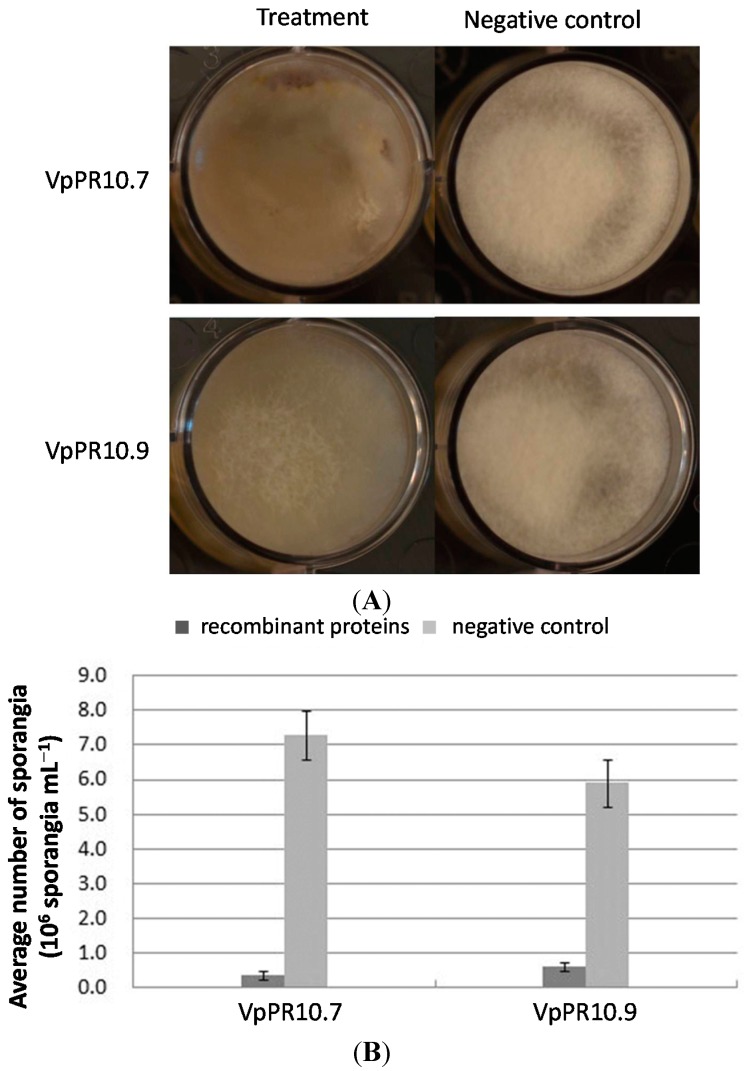
*In vitro* anti-fungal activities of recombinant VpPR10s proteins. (**A**) Growth inhibition of *Botrytis cinerea* treated with VpPR10 proteins. **Left**: 30 μL *Botrytis cinerea* sporangial suspension + 100 μL purified recombinant proteins (treatment); **Right**: 30 μL *Botrytis cinerea* sporangial suspension + 100 μL glutathione buffer (negative control); and (**B**) Quantitative analysis of anti-fungal activities of VpPR10 proteins.

The recombinant proteins VpPR10.7 and VpPR10.9 were selected for the *in vitro* anti-fungal activity assay. Their inhibition of the growth of *B. cinerea* was shown in Figure 7. Preliminary screening of VpPR10.7 confirmed that it could strongly inhibit *B. cinerea* growth (Figure 7A) whereas VpPR10.9 showed only slight inhibition compared with the negative control (Figure 7A). Figure 7B showed a quantitative analysis of anti-fungal activities of VpPR10.7 and VpPR10.9. The average number ofsporangia showed big differences between treatment with VpPR10.7 and the negative control (Figure 7B). However, the average number of sporangia decreased less with VpPR10.9 (Figure 7 B). This result was identical with that in Figure 7A.

### 2.6. Influences of VpPR10s on E. coli under Abiotic Stresses

**Figure 8:**
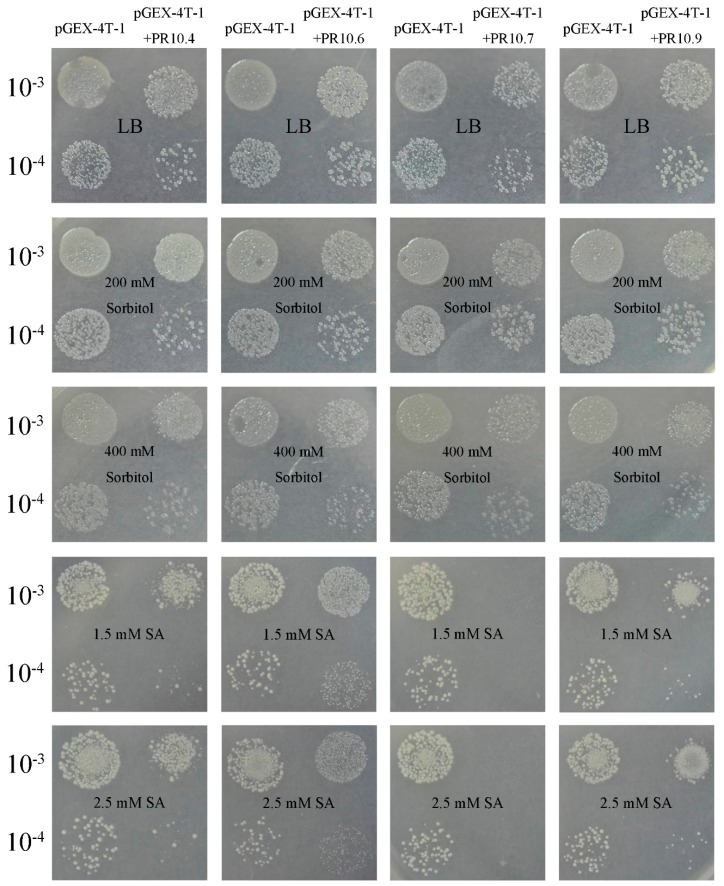
Growth analysis of *Escherichia coli* cells containing VpPR10s recombinant plasmid and pGEX-4T-1 alone in solid LB media with different supplements. Solid LB media and *E. coli* cells transformed with pGEX-4T-1 were used as controls.

The growth of *E. coli* cells was studied under different stress conditions. Solid LB media and *E. coli* cells transformed with pGEX-4T-1 were used as controls. Recombinant *E. coli* cells transformed with VpPR10.4 or VpPR10.7 had fewer colonies compared to controls transformed with pGEX-4T-1 in the presence of sorbitol and SA (Figure 8). However, the growth of *E. coli* cells showed no significant differences between recombinant cells transformed with VpPR10.6 or VpPR10.9 and controls transformed with pGEX-4T-1 on the LB plates supplemented with sorbitol and SA (Figure 8).

## 3. Discussion

Pathogenesis-related genes (*PR10*) exist widely in the genomes of higher plants. Most *PR10* genes have open reading frames (ORF) of 456–489 bp, which encode peptides containing 151–162 amino acid residues, with molecular masses of 15–18 kDa [54]. The work of Gao *et al.* (2005) confirms that the ORF of *PR10s* are usually inserted by a 76–359 bp intron—although one subtribe of the *Malus PR10* gene family has no introns in the ORF [55]. In this experiment, we show that the *VpPR10* sequences share the characteristics described above. Thus, *VpPR10.4*, *VpPR10.6*, *VpPR10.7*, *VpPR10.9* contain ORFs of 480, 450, 477 and 486 bp, which encode peptides of 159, 149, 158 and 161 amino acid residues, with molecular masses of 17.34, 16.90, 17.45 and 18.41 kDa, respectively. All these ORFs are inserted by the introns at 189/190 bp (*VpPR10.4* and *VpPR10.7*) or 183/184 bp (*VpPR10.6* and *VpPR10.9*). These results indicate that the introns have highly-conservative positioning and that the *VpPR10s* are typical members of the *PR10* family.

Most PR10 proteins contain a highly conservative P-loop domain and a Bet v 1 motif [35,38]. It has been reported that the P-loop domain and Bet v 1 motif are contained in the amino acid sequence of PR10 proteins in *Lupinus albus* (LaPR10) [40], Asiatic cotton (GaPR10) [36], peanuts [37] and pepper [42]. The relationships between the two conservative domains and nuclease activity were further confirmed in our experiment. The sequences of VpPR10.4 and VpPR10.7 amino acids contain characteristic amino acid sites with nuclease activity. These are E_102_, E_149_ and Y_151_, respectively. However, E_102_ is replaced by D_102_ in the sequence of VpPR10.9 and E_149_, Y_151_ are replaced by Q_149_, H_102_ in VpPR10.6 (Figure 1). Hence, it can be speculated that VpPR10.4 and VpPR10.7 proteins are likely to have nuclease activities and VpPR10.6 and VpPR10.9 proteins may not have this function.

In this study, there was an obvious degradation of “Baihe-35-1” total RNA when treated with VpPR10.4 or VpPR10.7 proteins (Figure 5A,C). Sequence analysis shows that amino acid sequences encoded by *VpPR10.4* and *VpPR10.7* contain conservative structure domains P-loop and Bet v 1 and characteristic amino acid sites (E102, E149 and Y151) with nuclease activity. The corresponding relations can be further verified among the conservative structure domains P-loop and Bet v 1, characteristic amino acid site of nuclease and RNase activities of proteins *in vitro*. The “Baihe-35-1” total RNA showed no degradation when treated with VpPR10.6 or VpPR10.9 proteins (Figure 5B,D). Sequence analyses indicate that amino acid sequences encoded by *VpPR10.6* and *VpPR10.9* have no P-loop domain or Bet v 1 motif.

The PR10.2 protein obtained from *V. pseudoreticulata* has both RNase and DNase activities [45]. This demonstrates that VpPR10 proteins possess DNase activity. The study of Agarwal *et al.* (2013) shows that JcPR-10a protein from *Jatropha curcas* exhibits simultaneous RNase and DNase activities [56]. It has also been confirmed that the VpPR10.1 in *V. pseudoreticulata*, a member of the PR10 family, is related to programmed cell death and DNA degradation when incubated with tobacco BY-2 suspension cells [57]. RNase activity of pea PR10.1 protein has been demonstrated [44]. In rice plants, PBZ1 protein, a PR10 family protein, also exhibits RNase activity [43]. Therefore, it can be confirmed that PR10 proteins in many plants have nuclease activity. The purified VpPR10.4 and VpPR10.7 proteins show not only RNase activity but also DNase activity (Figure 5 and Figure 6). This has important significance for studies of the biological activities of PR10 proteins in the defense responses of plants. We speculate that the P-loop domain and Bet v 1 motif may have relationships with the RNase and DNase activities of VpPR10 proteins.

It has been reported that some PR10 proteins have strong anti-fungal activities [37,41,49]. For example, VpPR10.2 protein displayed strong growth inhibition of *A. alternate* while over-expression of VpPR10.2 in *V. vinfera* strengthened resistance to *P. viticola* [45]. However, over-expression of STH-2, a member of the Ypr10 family, does not increase resistance of potato to *Phytophthora infestans* or to potato virus X [58]. These differences may be a result of selective inhibition by the PR10 proteins [37]. In the present study, VpPR10.7 in *V. pseudoreticulata* strongly inhibited the growth of *B. cinerea* (Figure 7), which indicates that VpPR10.7 with anti-fungus activity might play an important role in resistance to *E. necator* in the host plant. Meanwhile, VpPR10.9 protein slightly slowed the growth of *B. cinerea* (Figure 7).

The expressions of *VpPR10s* genes are induced with IPTG, so the growth of *E. coli* cells transformed with VpPR10s recombinant plasmids shows some variation under different abiotic stresses (Figure 8). Sorbitol causes osmotic stress to bacterial cells and ASA (Acetyl SA) inhibits DNA glycation by interacting with DNA [56]. The VpPR10.4 and VpPR10.7 proteins contain the P-loop domain and Bet v 1 motif, which relate to RNase and DNase activities. The growth inhibition of *E. coli* cells could be associated with the RNase activities of VpPR10.4 and VpPR10.7, as a result of RNA degradation. Thus, the accumulation of truncated mRNA could inhibit protein synthesis and hence slow bacterial growth. In this way, the cells would grow poorly when exposed to abiotic stresses (Figure 8). However, the VpPR10.6 and VpPR10.9 proteins have no DNase or RNase activities, so the growth of *E. coli* cells transformed with these two recombinant plasmids is unaffected when placed on LB plates with added sorbitol and SA (Figure 8). The same results have been confirmed in work by Parinita Agarwal *et al.* [56].

## 4. Experimental Section

### 4.1. Plant Material

Material of the Chinese wild grape *V. pseudoreticulata* accession “Baihe-35-1” was obtained from the grape repository of Northwest A&F University, Shaanxi, China.

Leaves of greenhouse-grown vines were harvested and immediately covered with aluminium foil, frozen in liquid nitrogen and stored at −80 °C pending use.

### 4.2. Full-Length Cloning of VpPR10s

Genomic DNA was extracted using the CTAB protocol [59]. Genomic DNA of “Baihe-35-1” was used as the template, to design two pairs of specific primers based on *VvPR10s* sequences, and amplified twice to obtain the sequences *VpPR10.4*, *VpPR10.6*, *VpPR10.7* and *VpPR10.9* using overlap extension PCR*.* All primers are listed in Table 1.The primers F1 + R2 and F2 + R1 were chosen for the first amplification. For each gene, two purpose fragments were recycled on 1% agarose gel electrophoresis, including upstream and downstream sections of intron. The second amplification was done using the mixture of two fragments as templates and primers F1 and R1. The amplification products were analysed by electrophoresis and the single-purpose fragment was recycled with the Universal DNA Purification Kit (Tiangen biotech, Beijing, China). The recycled products were ligated into pMD-19T vector, transformed into *Escherichia coli* Top 10 and screened on LB plates containing 100 mg·L^−1^ ampicillin using blue-white selection.

**Table 1:** Primers for gene splicing by overlap extension PCR.

Primer Name	Sequence (5' to 3')
*VpPR10.4F1*	5'-ATGGGTGTTATTACTTATGAGATG-3'
*VpPR10.4R1*	5'-TTAATAAGCATCAGGATTTGCCAAG-3'
*VpPR10.4F2*	5'-CTACTTTGGTGAAGGTCACCAATTCAAGAGCGTGACACA-3'
*VpPR10.4R2*	5'-CACGCTCTTGAATTGGTGACCTTCACCAAAGTAGATC-3'
*VpPR10.6F1*	5'-ATGGGTGCTATCACTTATGAAATGGA-3'
*VpPR10.6R1*	5'-TTAATAGGCATCAGGATTGGCCAAG-3'
*VpPR10.6F2*	5'-GATTACTTTCGGTGAAGGCAGCCAATTCAACTACGTG-3'
*VpPR10.6R2*	5'-GTTGAATTGGCTGCCTTCACCGAAAGTAATCTTCTTG-3'
*VpPR10.7F1*	5'-ATGGGTGTTGTCACTTACACTG-3'
*VpPR10.7R1*	5'-TCAGGCATCAGGATTAGCTAAGAG-3'
*VpPR10.7F2*	5'-GAACTTTGCTGAAGGCTACCAATTCAAATATGTGAAGC-3'
*VpPR10.7R2*	5'-GCTTCACATATTTGAATTGGTAGCCTTCAGCAAAGTTCA-3'
*VpPR10.9F1*	5'-ATGGGTGTCACAAGACTCAGT-3'
*VpPR10.9R1*	5'-TCAAGTATAGGCGCGAGGGTGT-3'
*VpPR10.9F2*	5'-CAGATCAACTTCACTGAAGCTAGTCCTTTAACATACATG-3'
*VpPR10.9R2*	5'-GTATGTTAAAGGACTAGCTTCAGTGAAGTTGATCTGTTTG-3'

### 4.3. Sequence Alignment of VpPR10s

Splicing sequences were used for homology analysis with the online BLAST program (http://www.ncbi.nlm.nih.gov/BLAST) [60]. Sequence alignments of nucleotides and amino acids were analysed using DNAMAN software and the evolutionary tree of amino acid sequence was designed using MEGA4.0.

### 4.4. Prokaryotic Expression of VpPR10s and Purification of Recombinant Proteins

Based on the *VpPR10s* sequences, two primers were designed with the flanking restriction sites of *EcoR1* in the forward primer and *Sal1* in the reverse primer (*VpPR10.4*, *VpPR10.6*, *VpPR10.7*), while the *EcoR1* was replaced by *BamH1* in VpPR10.9. The complete coding sequences of *VpPR10s* were amplified using PCR carried out at an annealing temperature of 58 °C for 35 cycles. The amplified products were ligated into the cloning vector pMD19-T and transformed into *E. coli* Top 10. The selected recombinant cloning vectors pMD19-T/*VpPR10s* were digested with *EcoR1* (*BamH1*) and *Sal1*, and the *VpPR10s* gene segments were sub-cloned into the expression vector of pGEX-4T-1. The recombinant plasmid was transformed in *E. coli* (BL21) strains and grown in LB medium at 37 °C to OD_600_ 0.8. The VpPR10s proteins were induced with 0.1 mM IPTG and bacterial cells were harvested after induction at 30 °C for 4 h. The expression and purification of recombinant proteins were carried out using the methods described by Xu *et al.* [61]. Fusion proteins were purified with GST resin by affinity chromatography. A 1 mL volume of GST resin was centrifuged at 4 °C for 5 min at 7000 rpm, then 8 mL precooling PBS was added and mixed well with the resin. The treated GST resin was kept on ice pending use. The induced bacterial cells were lysed for 10 min at room temperature and then centrifuged for 5 min at 7000 rpm. The supernatant was transferred to cool GST resin and incubated at 4 °C for 30 min at 50 rpm to ensure GST tag proteins were fully bound to the GST resin. Next, 8 mL cool PBS was added and gently inverted to elute the free proteins. GST resin was then added with 800 μL reduced glutathione buffer and incubated at 4 °C for 30 min at 50 rpm to elute the bound proteins. The recombinant proteins were then collected by centrifuging at 4 °C for 5 min at 7000 rpm. The pGEX-4T-1 empty vector in BL21 was used as control.

### 4.5. DNase and RNase Activities Assays of Recombinant Proteins

Two gradient experiments were performed to investigate the effect of protein concentration on nuclease activity of VpPR10.7 recombinant protein. The influence of protein concentration on RNase activity was determined using the reaction systems included 100 ng·μL^−1^ “Baihe-35-1” leaf total RNA mixed with 0, 2, 4, 6, 8 or 10 μg VpPR10.7 recombinant protein. The total volume of the system was 20 μL. The mixture was thoroughly incorporated and held at 37 °C in a water bath for 30 min. Thereafter, 20 μL chloroform was added to these systems to terminate the responses. The mixture systems were held on ice for 10 min and 1% agarose gel electrophoresis was used to detect RNase activity. To determine the effect of protein concentration on DNase activity, 100 ng·μL^−1^ “Baihe-35-1” genomic DNA and 2.5 mM MgCl_2_ were incubated with 0, 2, 4, 6, 8 or 10 μg recombinant protein in reduced glutathione buffer for 30 min at 37 °C. The subsequent processes were identical. The effect of Mg^2+^ on DNase activity was also determined using two reaction systems with or without 2.5 mM MgCl_2_. All of the reaction temperatures and times were identical.

To determine the RNase activities of the purified recombinant proteins, Leaf total RNA was mixed with reduced glutathione buffer (negative control), boiled VpPR10s proteins (negative control) and the VpPR10s proteins (VpPR10.4, VpPR10.6, VpPR10.7, VpPR10.9). The total volume of the system was 20 μL. Each treatment contained 8 μg recombinant protein and the final concentration of RNA was 100 ng·μL^−1^. The mixture was thoroughly incorporated and held at 37 °C in a water bath for 30 min. Thereafter, 20 μL chloroform was added to these systems to terminate the responses. The mixture systems were held on ice for 10 min and 1% agarose gel electrophoresis was used to detect RNase activity.

To determine the DNase activities of the purified recombinant proteins VpPR10.4, VpPR10.6, VpPR10.7 and VpPR10.9, “Baihe-35-1” genomic DNA and MgCl_2_ were mixed with reduced glutathione buffer (negative control), boiled VpPR10s proteins (negative control) and the VpPR10s proteins (VpPR10.4, VpPR10.6, VpPR10.7, VpPR10.9). Each reaction included 8 μg of recombinant protein. Final concentrations of gDNA and MgCl_2_ were 100 ng·μL^−1^ and 2.5 mM, respectively. The subsequent processes were identical.

### 4.6. In Vitro Anti-Fungal Activity Assay

*In vitro* anti-fungal activity assays were conducted using the purified recombinant VpPR10s proteins. The fungus *Botrytis cinerea* was activated by incubating on potato dextrose agar (PDA) plates at 28 °C for four days and one colony (diameter 1 cm) was separated and suspended in sterile water. The sporangial suspensions contained the same amounts of sporangia (1.2 × 10^6^ sporangia·mL^−1^). A volume of 30 μL of sporangial suspension was mixed with 100 μL of each recombinant VpPR10s protein. The mixtures were added to the surface of solid PDA media and then dried and cultured at 28 °C for 5 days. Volumes of 30 μL of *B. cinerea* sporangial suspension and 100 μL of glutathione buffer were used as negative controls. When the five day culture period was complete, the *B. cinerea* on the surface of the solid PDA medium was removed and suspended in sterile water. The average number of sporangia (SN) per treatment was assayed using a haemocytometer and bright-field microscopy.

### 4.7. Functional Analysis of VpPR10s Proteins under Different Abiotic Stresses

Spot assays were carried out to investigate the abiotic stress responses of *E. coli* BL21 cells, transformed with VpPR10s recombinant plasmids and vector alone. *E. coli* cells were grown in liquid LB medium to OD_600_ 0.6. Next, 1 mM IPTG was added and cells were grown at 37 °C for 12 h. The induced *E. coli* cells were diluted to OD_600_ 0.6, and then diluted to 10^−3^ and 10^−4^. A volume 10 μL of each dilution was then spotted onto LB basal plates as controls and LB plates supplemented with 200 mM sorbitol, 400 mM sorbitol, 1.5 mM SA and 2.5 mM SA. The experiment was repeated three times.

## 5. Conclusions

In conclusion, our analysis of protein structure indicated that VpPR10.4 and VpPR10.7 had the P-loop domain and the Bet v 1 motif, which were consistent features of plant PR10. However, there was no P-loop domain or Bet v 1 motif in VpPR10.9 and we could not find the Bet v 1 motif in VpPR10.6. The results also demonstrated that VpPR10.4 and VpPR10.7 proteins had obvious RNase, DNase and anti-fungal activities in response to abiotic stress, while VpPR10.6 and VpPR10.9 proteins did not exhibit these behaviours.

## Abbreviations

ABA: abscisic acid
CTAB: cetyltrimethyl ammonium bromide
IPTG: isopropyl-β-d-thiogalactoside
JA: jasmonic acid
PDA: potato dextrose agar
PR: pathogenesis-related
SA: salicylic acid
SDS-PAGE: Tsodium dodecyl sulfate polyacrylamide gel electrophoresis
Vp: *Vitis pseudoreticulata*
